# Effect of drug dose and timing of treatment on the emergence of drug resistance *in vivo* in a malaria model

**DOI:** 10.1093/emph/eoaa016

**Published:** 2020-06-01

**Authors:** Mónica M Acosta, Joshua T Bram, Derek Sim, Andrew F Read

**Affiliations:** e1 Department of Biology, Center for Infectious Disease Dynamics, Pennsylvania State University, University Park, PA 16802, USA; e2 Department of Entomology, Pennsylvania State University, University Park, PA 16802, USA

**Keywords:** drug resistance, *Plasmodium chabaudi*, drug treatment timing, *in vivo*, *de novo*, evolution, population size, drug dose

## Abstract

**Background and objectives:**

There is a significant interest in identifying clinically effective drug treatment regimens that minimize the *de novo* evolution of antimicrobial resistance in pathogen populations. However, *in vivo* studies that vary treatment regimens and directly measure drug resistance evolution are rare. Here, we experimentally investigate the role of drug dose and treatment timing on resistance evolution in an animal model.

**Methodology:**

In a series of experiments, we measured the emergence of atovaquone-resistant mutants of *Plasmodium chabaudi* in laboratory mice, as a function of dose or timing of treatment (day post-infection) with the antimalarial drug atovaquone.

**Results:**

The likelihood of high-level resistance emergence increased with atovaquone dose. When varying the timing of treatment, treating either very early or late in infection reduced the risk of resistance. When we varied starting inoculum, resistance was more likely at intermediate inoculum sizes, which correlated with the largest population sizes at time of treatment.

**Conclusions and implications:**

(i) Higher doses do not always minimize resistance emergence and can promote the emergence of high-level resistance. (ii) Altering treatment timing affects the risk of resistance emergence, likely due to the size of the population at the time of treatment, although we did not test the effect of immunity whose influence may have been important in the case of late treatment. (iii) Finding the ‘right’ dose and ‘right’ time to maximize clinical gains and limit resistance emergence can vary depending on biological context and was non-trivial even in our simplified experiments.

**Lay summary:**

In a mouse model of malaria, higher drug doses led to increases in drug resistance. The timing of drug treatment also impacted resistance emergence, likely due to the size of the population at the time of treatment.

## INTRODUCTION

There is widespread agreement that appropriate antimicrobial use is critical for minimizing the emergence and spread of antimicrobial resistance. This belief is encapsulated in the mantra that resistance development can be inhibited by the right drug at the right time at the right dose for the right duration [1, 2]. Here, we focus on two of those factors—timing and dose—and ask experimentally how they affect the emergence of *de novo* drug resistance in an animal model. Our goal is to further advance the underlying science needed to identify treatment regimens which minimize resistance emergence while maximizing host health. Surprisingly few experimental studies have addressed the issue *in vivo* [3, 4].

The *de novo* evolution of drug resistance poses a significant challenge for the management of many diseases [5, 6]. In some cases, *de novo* resistance contributes to treatment failure, as with cancer [7] and several quickly evolving viral [8–10] and bacterial infections [11–13]. In other cases, *de novo* evolution is important as a source of resistance that is then transmitted and spread within a population [14]. For problematic *de novo* resistance evolution to occur in a patient, two conditions must be satisfied. First, resistant variants must appear, either by mutation or, in the case of some bacteria, by horizontal gene transfer. Second, resistant sub-populations must then expand to densities within their host that trigger symptoms and/or become transmissible. Both of these processes are impacted by drug treatment.

A common belief is that high enough drug doses can prevent resistance [15–20] because higher drug concentrations are more likely to kill resistant mutants [15, 21, 22]. In the limit, this is clearly true, but if concentrations sufficient to kill all resistant mutants cannot be achieved, populations of surviving mutants rapidly expand to fill the niche vacated when pathogens are killed by chemotherapy [3, 4, 23–25]. This process of niche expansion (also called ‘competitive release’) has been demonstrated in animal disease models [24, 26–28] and in humans [29, 30]. Competitive release means that in the simplest case, the relationship between drug dose and resistance emergence is an ‘inverted U’: at very low doses, there is no selection for resistance, at high doses, everything is killed, and in between, resistance evolution is promoted [3, 4, 31]. The inverted U has been frequently observed *in vitro* [4], but there has been very limited *in vivo* testing [3, 4]. *In vivo* testing is important for at least three reasons. First, immunity is a key determinant of both antimicrobial efficacy and pathogen population sizes [4, 32–36]. Second, within-host density-dependent population regulation is a key determinant of competition between wildtype and mutant populations [26, 37]. Third, and perhaps most important, resistance-minimization strategies have to be considered in the context of patient health, which by definition can be directly measured only *in vivo*.

Another common belief is that the earlier treatment begins, the less likely resistance is to arise [21, 38, 39]. The thinking is that adaptive evolution (here resistance) proceeds faster in larger populations because larger populations are more likely to contain mutations on which selection can act [40–43]. This is well verified *in vitro*, where resistance is more likely to emerge when larger populations are treated with antimicrobials [42]. However, theoretical analyses have pointed to important complexities that can arise *in vivo* [32–34, 44, 45]. For example, density-dependent immune components may be differentially affected by pathogen exposure, which early treatment can truncate, restricting control of resistance in the context of developing immunity [34]. Furthermore, increases in population size do not always result in increased levels of genetic diversity. For example, small differences in the growth rate between resistant mutants and wild type parasites can reduce genetic diversity over time [46]. The few *in vivo* tests of which we are aware [38, 39, 47–50] involved early or late drug treatment or infections initiated with small or large inocula [39, 49]. In these binary cases, resistance typically emerged less readily if infections were treated early or had fewer pathogens to begin with, consistent with the idea that larger populations are more likely to contain resistant mutants.

Here, we use a rodent model of malaria to test the effects of drug dose and timing of treatment (day post-infection) on the *de novo* emergence of resistance. To do so, we use the antimalarial atovaquone, a highly effective antimalarial drug when used in combination with proguanil, but that when used as a monotherapy results in treatment failure in up to 30% of patients due to resistance evolution [51, 52]. Atovaquone works by inhibiting the ability of parasites to maintain their mitochondrial membrane potential [53] and by disrupting the regeneration of ubiquinone, a critical step in parasite pyrimidine synthesis [54]. Single point mutations in the mitochondrial *cytochrome b* (*cytb*) gene, however, have been found to confer high-level resistance in both animal [51, 55–57] and human models [58]. We reasoned that a strong resistance phenotype that readily evolves and is conferred by easily assayed genetic changes would make possible an experimental analysis of the impact of dose and timing of treatment on the probability of resistance emergence.

Previous experiments in a similar malaria model have shown that competitive release of resistant parasites is dose-dependent, with high drug concentrations promoting greater expansion of resistant populations [24, 27, 28]. In those experiments, resistant populations were seeded at known frequencies prior to drug treatment. We show here that competitive release of *de novo* resistance (on an isogenic background) is also dose-dependent when the probability of expected resistance is high as it is with atovaquone. Further, we use our model to explore the effect of timing of drug treatment on resistance emergence across a broader range of time periods than previously tested *in vivo*. Our results confirm that treatment early in infection (a ‘hit early’ strategy) reduces the likelihood of resistance, but we also find that very late treatment reduces the likelihood of resistance emergence. We interpret our findings in the context of effects of treatment regimen on host health and suggest that identifying the ‘right’ dose and the ‘right’ time may be non-trivial.

## METHODOLOGY

### Experimental overview

**Figure 1.**
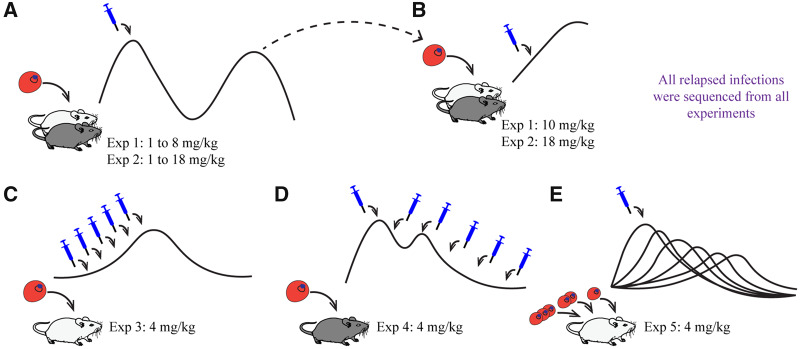
Experimental overview. Cartoon depictions show initiated infections with malaria infected cells in red, drug treatment with a syringe in blue and parasite dynamics through time as a black line. Black and white mice represent C57BL/6 and Swiss Webster mouse lab strains, respectively. (**A**) Effect of atovaquone dose and resistance emergence (experiments 1 and 2). Mice were drug treated with varying doses and monitored for relapse. A subset of relapsing infections was passaged to naïve mice (**B**) and drug-treated with a high dose to obtain a phenotypic measure of resistance (see Methods section). All relapsed infections were sequenced at the Qo2 region of the *cytb* gene for high-level resistance. (C-E) Experimental manipulations of timing of drug treatment (days post-infection) or inoculum size. In experiments 3 (**C**) and 4 (**D**), infections were treated at different times leading up to or following peak parasitemia, respectively. In experiment 5 (**E**), treatment day was fixed, but different inoculum sizes used to seed infections. Mice in experiments 3–5 were monitored for relapse and similarly genotyped for high-level resistance as experiments 1 and 2

We conducted five separate experiments (Fig. 1 and Table 1). In our first two experiments, we explored the effect of atovaquone dose on the probability of resistance emergence and treatment failure (Fig. 1A  and B). In experiment 1, groups of infected mice were treated with contrasting atovaquone doses. In experiment 2, we extended the range of doses and assessed patterns of resistance and treatment failure in a different strain of mouse to determine the generality of our results. In the next two experiments, we held atovaquone dose constant and looked at the impact of drug treatment timing on resistance emergence, treating either at various points prior (experiment 3) or following (experiment 4) peak parasite densities (Fig. 1C and D). As these experiments suggested population size was likely important, we conducted a final experiment (experiment 5) to directly vary population size at the time of treatment (Fig. 1E). In this last experiment, we manipulated the number of parasites used to initiate infections (inoculum size) and kept the timing of treatment constant.

**Table 1.** Experimental details

Experimental group	Inoculum size	Treatment start	Total mice	Total in analysis	No. relapsed	No. resistance phenotyping
Atovaquone dose						
Experiment 1 (C57BL/6) (mg/kg)						
0	10^6^	6	10	9^a^		
1	10^6^	6	10	10	10/10	2
2	10^6^	6	10	9^b^	8/9	3
4	10^6^	6	10	8^a^^b^	6/8	4
8	10^6^	6	10	9^b^	5/9	5
Experiment 2 (Swiss Webster) (mg/kg)						
0	10^6^	6	10	1^9a^		1
1	10^6^	6	10	8^a^^bc^	8/8	7
2	10^6^	6	10	9^a^	9/9	5
4	10^6^	6	15	12^2ab^	12/12	7
8	10^6^	6	20	17^2acd^	15/17	8
12	10^6^	6	20	15^5a2c^	15/15	7
18	10^6^	6	20	15^4abc^	14/15	7
Population size and timing						
Experiment 3 (Swiss Webster) (days PI)						
3	10^2^	3	7	7	0/7	
5	10^2^	5	7	6^a^	0/6	
7	10^2^	7	7	6^a^	0/6	
9	10^2^	9	7	7	2/7	
11	10^2^	11	7	6^b^	6/6	
Experiment 4 (C57BL/6) (days PI)						
6	10^6^	6	8	7^b^	7/7	
9	10^6^	9	8	4^4a^	2/4	
12	10^6^	12	8	6^2a^	2/6	
15	10^6^	15	8	7^a^	0/7	
18	10^6^	18	8	4^3ab^	0/4	
21	10^6^	21	8	6^a^^b^	0/6	
Experiment 5 (Swiss Webster) (inoculum)						
10^2^	10^2^	6	8	7^b^	2/7	*** ***
10^3^	10^3^	6	8	5^3b^	5/5	*** ***
10^4^	10^4^	6	8	7^b^	7/7	*** ***
10^5^	10^5^	6	8	7^bc^	6/7	*** ***
10^6^	10^6^	6	8	7^b2c^	7/7	*** ***
10^7^	10^7^	6	8	3^5a^	3/3	*** ***

All drug treatments were performed on two successive days. In the case of experiments 3–5, 4 mg/kg was used. Mice were omitted from analysis if they died during acute stage infection or were mis-inoculated (see Methods section). A phenotypic measure of resistance was only performed in experiments 1 and 2.

a Mice which died during the acute stage of infection,

b Mis-inoculated mice (defined in the text),

c Mice which died during relapse, but are included in the analysis except where indicated,

d Mouse which was omitted from analysis due to unobtainable Sanger sequencing data. Number immediately preceding subscript relates to number of mice in each category.

To avoid ambiguity in what follows, we restrict the word ‘treatment’ to mean drug treatment and use the terms experimental ‘group’ or ‘manipulation’ to refer to differences in the timing of drug treatment or inoculum size.

### Methods

#### Parasites and hosts

Parasites were of the AS_13p_ clone of *Plasmodium chabaudi* which has had no known prior exposure to atovaquone. This clone represents a lineage following from a direct cloning event (i.e. population expansion from one initial individual), but has since seen limited propagation in mice. We thus assume the population to be highly homogenous, but cannot exclude the presence of some genetic diversityin this population. All parasite inoculations into mice were performed intraperitoneally by diluting infected red blood cells in a total volume of 100 μl of citrate saline. Citrate saline was made by dissolving 8.5 g of sodium chloride and 15 g of tri-sodium citrate in 1 l of distilled water, autoclaved and pH adjusted to 7.2 using sodium hydroxide. All experimental mice were female, aged six to eight weeks old at the start of the experiments. Experiments 1 and 4 were conducted with C57BL/6 mice, while experiments 2, 3 and 5 involved outbred Swiss Webster mice (Table 1). These two strains of mice differ in terms of both genetic diversity and susceptibility to *P. chabaudi* infection. Swiss Webster mice represent an outbred, but highly susceptible strain while inbred C57BL/6 mice are known instead as a resistant strain. These differences in sensitivity to infection likely arise due to a preferential Th1 biased immune response in C57BL/6 mice that leads to resolution of infection even in the absence of drug treatment [59]. We used two different strains of mice across our experiments for two reasons: (i) to determine the sensitivity of resistance emergence across doses to differences in host genetic diversity and susceptibility to malaria infection and (ii) in the case of experiment 4, because treatment late in infection warranted the use of resistant C57BL/6 mice due to high probabilities of mortality in Swiss Webster mice if treatment was delayed until periods post-peak infection. Mice were housed in cages of four or five individuals and maintained on a diet of 0.05% para-aminobenzoic acid (an organic compound added to facilitate parasite growth in this model [60]) and on mouse chow (PicoLab^®^ Rodent Diet 20).

#### Drug treatment

The bioavailability of atovaquone is known to be highly variable and low given orally administered doses [61]. As such, we chose to dissolve atovaquone in DMSO and administer all doses via intraperitoneal injection. Such a method has also been previously used in other rodent malaria models [55–57]. In these models (predominantly *P. berghei* infections in BALB/c mice), inhibition of parasite replication has been observed at doses as low as 0.04 mg/kg (intraperitoneal injection) [62] with resistance emergence at 5 mg/kg (single oral dose delivery for two consecutive days) [51] and 14.4 mg/kg (total intraperitoneal dose given once per day for 1–3 days) [57]. We chose drug doses spanning these values (1–18 mg/kg) and chose to treat for 2 days to both ensure we would observe resistance emergence (to enable quantification), and in expectation of reaching curative doses. Atovaquone was dissolved and diluted in DMSO and up to 100 μl was inoculated intraperitoneally on the morning of days of drug treatment.

#### Monitoring infections

In most cases, mice were monitored daily starting 3 days post-infection. Exceptions were experiment 2, in which mice were sampled only on even days starting 4 days post-infection, and during periods late in infection in experiments 3–5, when sample frequency was changed to every 2 or 3 days. Details of blood sampling, quantification of red blood cell (via coulter counter) and parasite densities (quantitative PCR) are described elsewhere [63, 64].

#### Genotyping for resistance

As genetic markers of resistance, we focused on the development of high-level resistance, which is associated with SNP mutations in the Qo2 domain of the parasite’s mitochondrially encoded *cytb* gene [55, 56, 58, 62]. As such, we amplified and Sanger sequenced a 422 base pair region encompassing the entire Qo2 region (Supplementary Fig. S1). Samples were analyzed and aligned to an available reference sequence of *cytb* for *P. chabaudi* strain AS (GI: 222425464) using Geneious^®^ version 9.1.8. Lower level resistance is associated with SNPs in other domains of the *cytb* gene [57, 62], but we focus on mutations in the Qo2 domain because slowing and even preventing the emergence of high-level resistance is a long-term goal of evolutionary medicine [4]. Thus, our study focuses on the impact of contrasting treatment regimens on the emergence of resistance encoded in the Qo2 domain. While some of the parasite populations subjected to drug treatment in our experiments may have acquired mutations in genic regions other than the Qo2 domain (which we did not assay for), we refer to Qo2 wildtype parasite as atovaquone sensitive for ease of discussion while acknowledging that some degree of resistance could have been provided by other mutations. Further details regarding our genotypic sequencing are available in the Supplementary Material. A phenotypic measure of resistance was included in our experiments in which we varied dose, which we detail below.

### Experimental details

#### Drug dose and emergence of resistance

For experiments 1 and 2 (C57BL/6 and Swiss Webster mice, respectively), hosts were infected with 10^6^ parasites and drug treated on days 6 and 7 post-infection when parasite densities peak and mice become lethargic, anemic and lose weight. Drug doses spanned 1 to 8 mg/kg and 1 to 18 mg/kg for each experiment, respectively (Table 1 and Fig. 1A). To obtain a phenotypic measure of resistance, a subset of relapsed infections in all experimental groups (sampled haphazardly, with a goal of capturing at least three per group) were passaged to naïve mice (infections initiated with 10^6^ parasites, Table 1 and Fig. 1B). These were then drug treated (10 or 18 mg/kg, experiments 1 and 2, respectively) on days 3 and 4 post-infection and monitored until day 7 post-infection. All relapsed infections were sequenced as detailed above.

#### Timing of treatment, population size and emergence of resistance

In experiments 3–4, we kept drug treatment constant at 4 mg/kg for two successive days and varied the timing of drug treatment by treating at points prior to or following peak parasite densities, when parasite populations are growing or shrinking (Table 1 and Fig. 1C and D). In experiment 3, we infected Swiss Webster mice with 100 parasites and drug treated at various times before peak parasitemia. Inoculation with 100 parasites results in prolonged time to maximum parasitemia [65] and we choose this parasite dose to maximize the number of possible drug treatment periods following inoculation and prior to peak densities. In experiment 4, we infected resilient C57BL/6 mice with 10^6^ parasites, and drug treated on different days after peak parasitemia (few Swiss Webster mice survive peak parasitemia in the absence of drug treatment). Finally, in experiment 5, we infected Swiss Webster mice with different numbers of parasites (10^2^, 10^3^, 10^4^, 10^5^, 10^6^ or 10^7^) and drug treated on days 6 and 7 post-infection. All relapsed infections were sequenced as detailed above, and in this case a phenotypic measure of resistance was not assayed.

#### Definitions of parasite relapse and resistance

Relapse is the appearance of sustained parasite populations after drug treatment. In what follows, we use the terms relapse and treatment failure interchangeably. We operationally defined this as parasite densities above our assay detection threshold for more than three consecutive sampling time points. In all cases, relapse was easily identifiable, and parasites remained above detectable levels for at least 6 days. Resistance was defined as relapse that resulted in any majority genotype that differed from the wildtype *cytb* sequence at the Qo2 domain. All mutations we found had been previously associated with atovaquone resistance or were at positions in which amino acid substitutions have previously been reported to result in resistance [51, 55, 57]. As such, our primary measures of relapse and resistance were binary. However, in experiments 1 and 2 we also obtained a continuous phenotypic measure of resistance, where we evaluated resistance as the ability for passaged parasites to grow in the presence of atovaquone in naïve mice.

#### Definitions of host health and other infection metrics

To quantify differences in host health across experimental manipulations, we calculated the lowest value for red blood cell densities early in infection (acute stage: days 3–13 post-infection) and late in infection (chronic stage: day 14 to end of monitoring) for each experiment. To determine differences in efficacy of drug treatment, we calculated the rate at which parasites declined during drug treatment (parasite clearance) by fitting individual linear models for each mouse on days 6–10 for experiments 1 and 2 and for four consecutive days following drug treatment for experiments 3–5. In the case of early treatment in experiment 3, or very late treatment in experiment 4, we were not able to calculate clearance rate, due to parasite densities being below qPCR detection rates. In experiments 1 and 2 where we passaged relapsed parasites to naïve mice, we calculated a phenotypic measure of resistance by fitting individual linear models for each mouse on days 3–6 post-infection.

#### Statistical analyses

All analyses and graphics were conducted in R, version 3.5.1 [66] and RStudio [67]. Parasite numbers per mouse were log10 transformed prior to all analyses. In all analyses where drug dose was used, dose was log10 transformed and treated as a continuous variable. Mice that had to be euthanized before relapse was possible were excluded from all analyses. Mice for experiments 1 and 2 which were under inoculated with parasites, defined as those with two log_10_ intervals lower than average parasite densities on the first day of monitoring, were similarly omitted from analyses. In the case of experiments 3–5, where population size on day of treatment was the focus, we were more conservative in our definition of mis-inoculations, dropping any mice which differed over a single log_10_ interval from the average parasite number on the first day of treatment (with the exception of mice which were treated on days 3 or 6 post-infection in experiment 3 which were below the level of detection at this timepoint).

Logistic regression was used to analyze probabilities of relapse and resistance. In the case of experiments 1 and 2, we modeled the probability of relapse and resistance separately as a function of dose, assuming all mice in each experimental group received equal doses of atovaquone (we did not measure actual serum concentrations). In the case of experiments 3–5 where we were interested in the effect of pathogen population size, we modeled the probability of relapse and resistance separately as a function of both experimental group and population size at the time of drug treatment. Pathogen population sizes were obtained from qPCR data and were unique to each mouse. In experiment 3, parasite numbers at the time of drug treatment were at times below our qPCR detection threshold when treated very early in infection. We estimated parasite numbers at the time of treatment for these mice by using estimates from a three-parameter logistic model fit to data from untreated mice (Supplementary Fig. S2). We used a generalized least squares model to analyze differences in pathogen population size across experiments using the R function VarIdent assuming different variances per experiment within the gls package.

We used linear models to analyze differences in passaged parasite growth, parasite clearance and minimum red blood cell densities during the acute and chronic stages of infection across experimental groups. In experiments 1 and 2, these metrics were modeled as a function of continuous log10 transformed dose. In experiments 3–5, we instead treated experimental manipulations as discrete groups in our analysis. All *P* values are for two-tailed tests.

## RESULTS

### Atovaquone dose

Our first two experiments tested the effect of atovaquone dose on resistance emergence (Fig. 1A and B).

**Figure 2.**
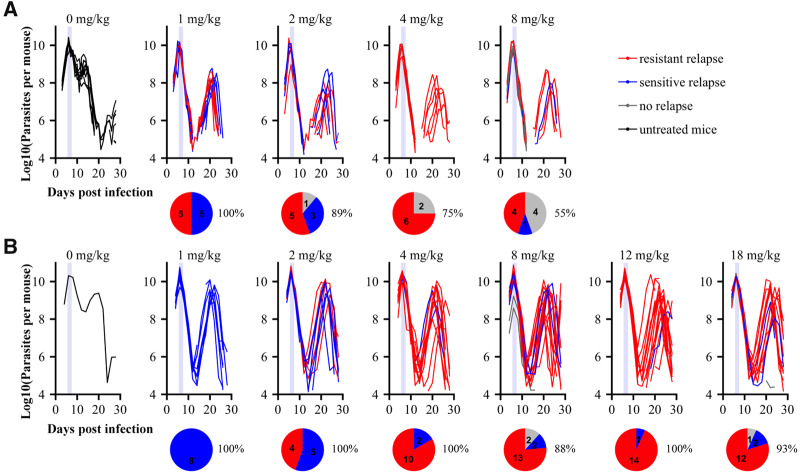
Parasite dynamics for individual mice in experiments 1–2 showing treatment failure and resistance. Parasite dynamics are shown through time for C57BL/6 and Swiss Webster mice in experiments 1 (**A**) and 2 (**B**), respectively. Red and blue indicate the identity of relapsing parasite populations from Sanger sequencing of the Qo2 region of the *cytb* gene, with blue representing wildtype genotypes and red indicating the presence of mutation(s) known to encode high-level resistance. In grey are dynamics of non-relapsing infections. Pie diagrams show proportion and total numbers of mice in each category. Percentages show proportion of total infections which relapsed per treatment. Dynamics of surviving mice which were not drug treated are shown in black. Drug treatment timing is represented as light purple bars

**Figure 3.**
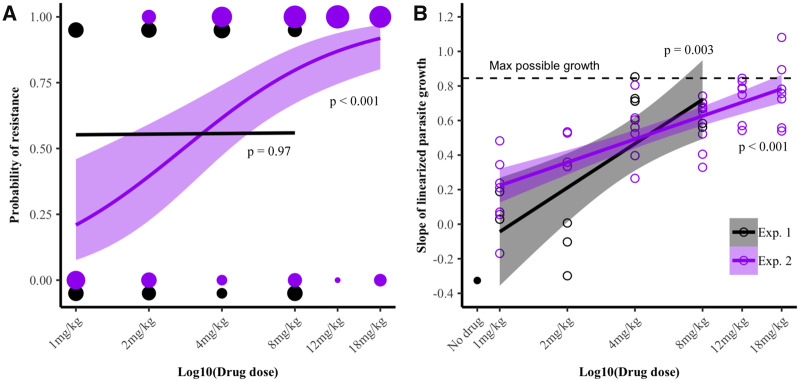
Dose-dependent emergence of atovaquone resistance. Plotted data from C57BL/6 and Swiss Webster mice for experiments 1 (black) and 2 (purple), respectively. (**A**) Relapsing populations after drug treatment were genotyped at the Qo2 region of the *cytb* gene and classified as wildtype (0) or drug-resistant (1). Probability of resistance evolution (mice with relapsing resistant infections out of all mice in each treatment) are plotted. Size of circles correspond to number of mice. Lines are from logistic regression models fitted for each experiment. (**B**) A subset of relapsing infections was passaged to naïve mice and atovaquone-treated. Plotted points show population growth rates in mice. Lines represent linear models of dose against parasite growth for each experiment. The dotted line represents the growth rate calculated for a burst size of seven merozoites per infected red blood cell (representing an average burst size for *P. chabaudi*). Error widths for lines shown are 95% confidence intervals

Following drug treatment on days 6 and 7 post-infection when parasite numbers are peaking, mice experienced both treatment failure (relapse) and resistance emergence in both experiments (summarized in Supplementary Table S1 and Fig. 2). In experiment 1 (C57BL/6 mice), treatment failure in general was prevented at higher doses, with fewer instances of relapse (relapse–log10 dose: *χ*_1,35_^2^ = 7.1, *P* = 0.01, Fig. 2A). However, resistance mutations in the Qo2 domain were as likely to emerge at any drug dose (resistance–log10 dose: *χ*_1,35_^2^ = 0.0, *P* = 0.97, Figs 2A and 3A). In contrast, in experiment 2 (Swiss Webster mice, wider range of drug doses), treatment failure occurred in almost all cases and was unrelated to drug dose (relapse–log10 dose: *χ*_1,75_^2^ = 1.2, *P* = 0.27, Fig. 2B), but the failures at higher doses were more likely to be due to resistance evolution (resistance–log10 dose: *χ*_1,75_^2^ = 19.7, *P* < 0.001, Figs 2B and 3A). When we limited our analysis to doses that were common between the two experiments (1–8 mg/kg), we found that the impact of dose on the probability that resistant mutants emerged differed between experiments (log10 dose × experiment: *χ*_1,79_^2^ = 7.1, *P* = 0.01).

To correlate our initial genotypic measure of resistance with phenotypic drug susceptibility of relapsing infections, a subset of infections (see Methods section) were passaged to naïve mice and treated with a high dose of atovaquone for 2 days early in infection (using 10 or 18 mg/kg for experiments 1 and 2, respectively). A total of 14 (48% of total relapses) for experiment 1, and 41 infections (56% of total relapses) for experiment 2 were tested in this manner. Parasites that had been genotyped as resistant grew better in the presence of the drug than those genotyped as wildtype (phenotypic measure of resistance–Qo2 genotype: *F*_1,55_ = 44.5, *P* < 0.001). Indeed, in all but one case, growth rates were positive, in contrast to wildtype parasites (Supplementary Fig. S3). Furthermore, populations of parasites that had been previously exposed to higher doses of atovaquone grew better, and given previous exposure to the highest doses approached growth rates in naïve drug-treated mice similar to those of wildtype parasites in non-drug treated hosts (experiment 1 (phenotypic measure of resistance–log10 dose): *F*_1,13_ = 14.5, *P* = 0.003; experiment 2 (phenotypic measure of resistance–log10 dose): *F*_1,40_ = 59.1 *P* < 0.001, Fig. 3B). Thus, higher drug doses led to parasite populations that had higher levels of resistance.

The effects of dose on resistance emergence were not obviously related to parasite clearance during drug treatment. Drug treatment with any dose of atovaquone resulted in significant decline of parasite numbers, reducing populations by nearly an order of magnitude per day (Supplementary Fig. S4a). Parasites were cleared more rapidly in experiment 1 (C57BL/6 mice) than they were in experiment 2 (Swiss Webster mice) (clearance–experiment: *F*_1,82_ = 24.3, *P* < 0.001). Rates of parasite clearance in drug-treated mice were unaffected by dose in experiment 1 (clearance–log10 dose: *F*_1, 35_ = 0.98, *P* = 0.3) and, unexpectedly, decreased with increasing dose in experiment 2 (clearance–log10 dose: *F*_1,76_ = 5.5, *P* = 0.02). Population sizes at the start of treatment were significantly larger in our second experiment, when using Swiss Webster mice (log10 population size–experiment: *F*_1,111_ = 65.0, *P* < 0.001, Supplementary Fig. S4b).

Drug treatment had no effect on the survival of C57BL/6 mice but rescued Swiss Webster mice (Supplementary Fig. S5). Among treated mice, however, dose did not affect survival (*P* = 0.4, *P* = 0.6 in experiments 1 and 2, respectively). We then assessed whether differences in dose manifested themselves in differences in anemia. To do so, we separately determined the lowest value for red blood cell densities (greatest degree of anemia) during the time frame of initial infection and drug treatment and during infection relapse following drug treatment. Higher doses did not limit anemia during either the acute (Supplementary Fig. S4c) or chronic (Supplementary Fig. S4d) stages of infection in either strain of mouse. These measures of anemia did differ significantly between strains of mice, however (acute stage minimum anemia–experiment: *F*_1,112_ = 23.6, *P* < 0.001; chronic stage minimum anemia–experiment: *F*_1,112_ = 58.0, *P* < 0.001), reflecting general differences in susceptibility to malaria infection (Supplementary Fig. S6).

### Timing of atovaquone treatment and parasite population size

**Figure 4.**
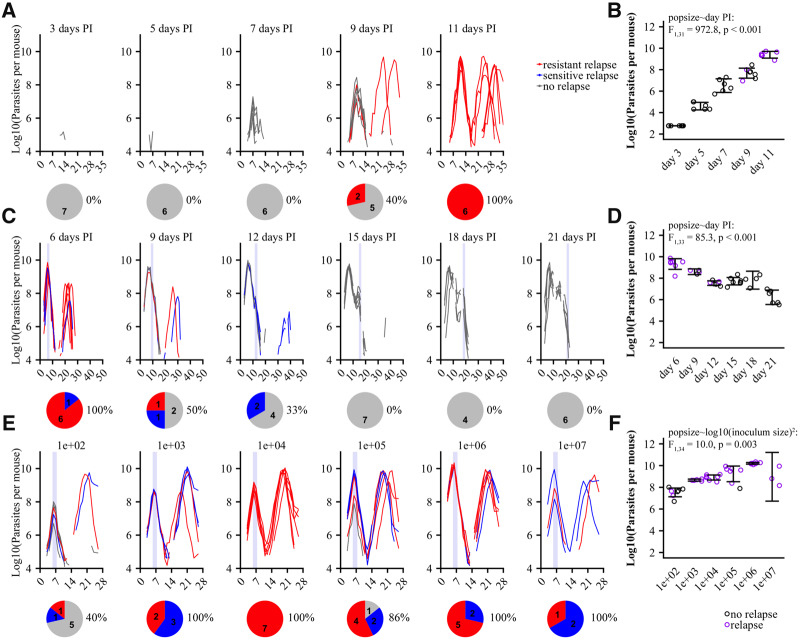
Individual parasite dynamics for experiments 3–5 and treatment failure and resistance. Left panels, parasite dynamics for all individual mice in experiments 3 (**A**), 4 (C) and 5 (E). Experiments 3 and 5 were performed with Swiss Webster mice while experiment 4 was performed with C57BL/6 mice. Colors indicate the consensus sequence of the Qo2 region of the *cytb* gene (wildtype, blue; resistance-associated mutants, red; grey, non-relapsing infections). Pie diagrams show proportion and total numbers of mice in each category. Percentages show proportion of total infections which relapsed per experimental group. Light purple bars, timing of drug treatment. Right panels, population sizes at time of drug treatment for experiments 3 (B), 4 (D) and 5 (**F**) for each experimental group; purple, infection relapsed; black, no relapse. Error bars represent 95% confidence intervals. Statistics demonstrate whether population size increased with time since inoculation (B and D) or with inoculum size (F)

In our second set of experiments (experiments 3–5), we held atovaquone dose constant and manipulated timing of treatment or inoculum size to assess the effect of timing and population size on infection relapse and resistance evolution (summarized in Figs 1C–E and 4 and Supplementary Table S1).

In experiment 3, Swiss Webster mice were infected with 100 parasites and treated at various times before peak parasitemia (Fig. 1C and Table 1). Resistance differed between experimental groups (resistance–experimental group: *χ*_4,31_^2^ = 27.6, *P* < 0.001) so that resistance only emerged in later treated infections (Fig. 4A). Later-treated infections had larger population sizes when treatment started (population size–day post-infection: *F*_1,31_ = 972.8, *P* < 0.001, Fig. 4B). Within experiment groups, there was variation in population size at time of treatment, but this variation did not additionally predict resistance emergence (*χ*_1,26_^2^ = 0.5, *P* = 0.47).

In experiment 4, C57BL/6 mice were infected with a million parasites and treated at various points after peak infection (Fig. 1D and Table 1). Treatment failure and resistance emergence differed between experimental groups (relapse–experimental group: *χ*_5,33_^2^ = 29.6, *P* < 0.001; resistance–experimental group: *χ*_5,33_^2^ = 24.3, *P* < 0.001), with treatment failure and resistance less likely in later-treated infections (Fig. 4C). Population sizes at the start of drug treatment were also smaller in the later-treated infections (population size–day post-infection: *F*_1,33_ = 85.3, *P* < 0.001, Fig. 4D). Within experiment groups, there was variation in population size at time of treatment, but this variation did not additionally predict the likelihood of relapse or resistance emergence (relapse: χ_1,27_^2^ = 1.3, *P* = 0.26, resistance: *χ*_1,27_^2^ = 0.03, *P* = 0.87).

In experiment 5, Swiss Webster mice were inoculated with different numbers of parasites to generate different populations sizes when drug treated on days six and seven post-infection (Fig. 1E, Table 1). Treatment failure and resistance differed among experimental groups, (relapse–experimental group: *χ*_5,35_^2^ = 18.3, *P* = 0.003; resistance–experimental group: *χ*_5,35_^2^ = 15.2, *P* = 0.01), with 100% of mice relapsing with resistant parasites at intermediate inoculum sizes. Here, population sizes at time of treatment did not continuously increase as inoculum sizes increased, reflected by a significant quadratic term in the relationship (population size–log10(inoculum size)^2^: *F*_1,34_ = 10.0, *P* = 0.003, Fig. 4F). Within experiment groups, there was variation in population size at time of treatment, but this variation did not additionally predict the likelihood of relapse or resistance emergence (relapse: *χ*_1,29_^2^ = 1.96, *P* = 0.16, resistance: *χ*_1,29_^2^ = 0.01, *P* = 0.94). Note that the variation in population size at time of treatment was substantially less in this experiment than in experiments 3 and 4 (*σ*^2^ = 0.92 vs *σ*^2^ = 1.17 for experiment 4 and *σ*^2^ = 5.81 for experiment 3, generalized least squares model allowing for variance structure, Supplementary Fig. S7).

**Figure 5.**
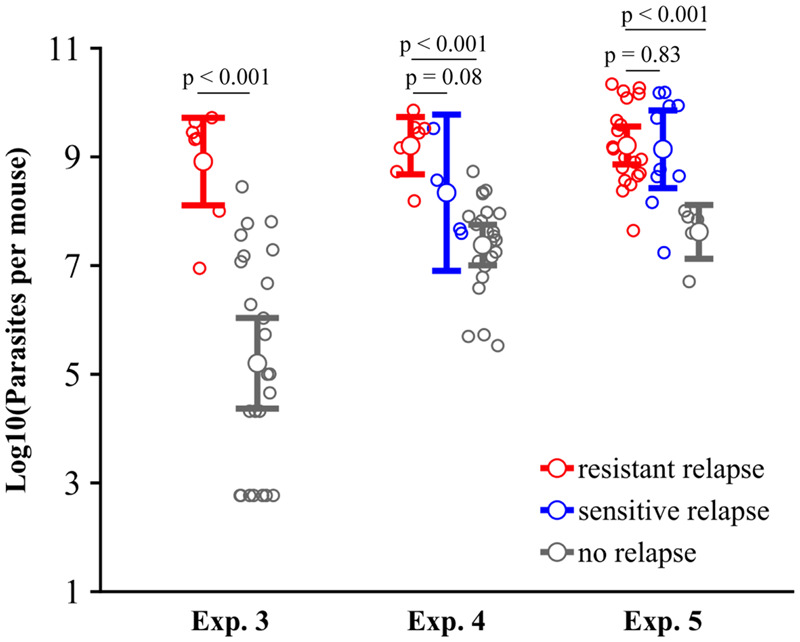
Treatment outcome as a function of parasite population size at the start of atovaquone treatment. Pathogen population sizes for non-relapsing (gray) infections, and for relapsing infections that were genotyped as drug-resistant (red) or wildtype/drug-sensitive (blue). In all three experiments, infections that relapsed had significantly larger parasite population sizes when treatment started, but resistant relapses were no more likely to emerge from larger populations than were sensitive relapses. Error bars are 95% confidence intervals

Thus, in all three experiments, relapse, resistance emergence and population size at the start of atovaquone treatment differed among experimental groups. We therefore asked if population size per se impacted relapse and resistance. Across all experiments, relapsing infections were characterized by larger population sizes at time of treatment (log10 population size–relapse in experiment 3: *F*_1,31_ = 25.8, *P* < 0.001; log10 population size–relapse in experiment 4: *F*_1,33_ = 24.0, *P* < 0.001; log10 population size–relapse in experiment 5: *F*_1,35_ = 20.2, *P* < 0.001, Fig. 5). Population size, however, did not determine whether those relapses were dominated by resistant or sensitive parasites (experiment 4: *F*_1,10_ = 3.9, *P* = 0.08; experiment 5: *F*_1,29_ = 0.04, *P* = 0.83, Fig. 5), but did increase the risk of resistance generally (Supplementary Fig. S8).

Differences in parasite clearance were observed across experimental groups in each experiment (clearance–experimental group in experiment 3: *F*_2,16_ = 22.3, *P* < 0.001; clearance–experimental group in experiment 4: *F*_4,25_ = 3.9, *P* = 0.02; clearance–experimental group in experiment 5: *F*_5,35_ = 3.7, *P* = 0.01; Supplementary Fig. S9a), but were not obviously related to patterns of resistance emergence. Early treatment reduced parasite densities less rapidly than later treatment in experiment 3 in Swiss Webster mice (Supplementary Fig. S9a), but resulted in less resistance (Fig. 4A). Similarly, larger parasite inoculums were cleared more rapidly in experiment 5 (clearance∼log10 population size at time of treatment: *F*_1,35_ = 19.8, *P* < 0.001, Supplementary Fig. S9a), but did not result in a concomitant decrease in resistance (Fig. 4E).

With respect to host health, differences in experimental group only affected survival in experiment 5 (Supplementary Fig. S10), and this was most obvious when mice were given large inoculum sizes (Supplementary Fig. S10c). Across experiments, early treatment (before peak parasite densities were reached in experiment 3) resulted in significantly less anemia than late treatment (experiment 4), even in more susceptible Swiss Webster mice (Supplementary Fig. S11a and b). Furthermore, in experiment 4, when mice were treated after peak parasite density, there was a tradeoff between treatment early in infection which generated more relapses, and treatment later in infection which resulted in more severe anemia early in infection (Supplementary Fig. S11b). We then assessed whether differences in experimental regimen manifested themselves in differences in anemia during the separate stages of acute and chronic infection. When mice were treated prior to peak parasite densities (experiment 3) or with differing inoculum sizes (experiment 5), experimental regimen impacted acute and chronic stage minimum red blood cell values (Supplementary Fig. S9b and c). Differences in timing of drug treatment following peak parasite density did not, however, affect these metrics (acute stage minimum anemia–experimental group: *F*_5,33_ = 2.0, *P* = 0.11; chronic stage minimum anemia–experiment: *F*_5,33_ = 1.7, *P* = 0.16, Supplementary Fig. S9b and c).

### Resistant mutants

In all cases, non-wildtype genotypes from relapsed populations contained mutations previously reported to result in high level resistance to atovaquone in *in vivo* experiments or from human field data [51, 55, 56, 58]. All detected mutations were confined to a short region of the Qo2 domain spanning only six amino acids in length (Supplementary Fig. S1). Various SNPs led to seven non-synonymous mutations (F267I/V, Y268C/N/S, L271V and K272R) resulting in a total of ten different consensus sequences across all five experiments (Supplementary Table S2). In most cases, consensus sequences reflected the presence of a single amino acid change, but in some instances, two mutations were found to co-occur in the consensus sequence. This was most often the case with mutations L271V and K272R, the other example being a single infection with mutations Y268C and L271V in experiment 2 (Supplementary Table S2). These are likely double mutant haplotypes because the mutations appear to be equally abundant as judged from the electropherogram, but we cannot exclude the possibility that these are mixtures of two populations each with single mutation haplotypes. Our data also suggested that in some cases there was parasite population diversity within single mice (Supplementary Material).

A large diversity of mutations was selected even within the context of a single dose across different mice. This is reflected in the proportion of mice which harbored parasites of various genotypes at different doses for experiments 1 and 2 (Supplementary Fig. S12a). While wildtype genotypes dominated relapsing populations at low doses, higher doses resulted in a greater proportion of mice harboring parasites of the Y268C genotype (proportion of resistant mutants with Y268C genotype–log10 dose: *χ*_1,5_^2^ = 24.1, *P* < 0.001). Moderate doses (8 mg/kg) resulted in the largest number of represented consensus genotypes within experiments 1 and 2, with a total of seven represented genotypes.

## DISCUSSION

Using an experimental system where *de novo* resistance readily emerges *in vivo*, we found that higher doses of atovaquone led to an increased risk of resistance in one experiment, and that higher level resistance (as reflected by our phenotypic measure of resistance) was increased in two. Timing of treatment (day post-infection) also impacted resistance emergence, and this appeared to be correlated with changes in population size. However, we were unable to completely exclude the influence of the immune response or other changes in the host environment that may have coincided with changes in population size. We discuss each of these results in turn.

### Dose and the emergence of resistance

When we varied atovaquone dose (experiments 1 and 2), increasing dose led to the evolution of increasingly higher-level resistance, as measured by the growth rates of relapsing parasites in drug-treated tester mice (Fig. 3B). Presumably high-level resistance emerges only at the highest drug doses because mutations conferring the greatest levels of resistance have accompanying costs that put parasites at a competitive disadvantage in the absence of strong drug pressure, meaning that these mutations can only emerge when parasites with mutations that confer lower-level resistance are killed. Indeed, both *in vivo* [57] and *in vitro* [68] studies have demonstrated that mutations in *cytb* that confer resistance to atovaquone not only result in fitness costs, but that these costs are dependent on the exact resistance haplotype. However, we know of no *in vivo* (or *in vitro)* assays of fitness costs that include all the resistance haplotypes that we observed in our data. Alternatively (or additionally), increasingly higher doses may have shifted the proportion of each relapsing population that was Qo2 resistant or wildtype, resulting in changes in our phenotypic measure of resistance. Future *in vitro* or *in vivo* tests of fitness costs from isolated resistant haplotypes would be useful in clarifying these hypotheses. We note, however, that fitness costs can be highly contextual and not easily identified under non-limiting conditions [69].

At the genetic level, the proportion of infections that harbored the Y268C mutant also increased with dose (Supplementary Fig. S12a). This mirrors the resistance phenotype results as the substitution of a tyrosine for a cysteine at position 268 is associated with one of the highest levels of resistance to atovaquone in both *in vivo* and *in vitro* studies [51, 55, 70]. However, the Y268C mutation was not the sole cause of the high-level resistance that emerged at higher doses. Indeed, other mutations were recovered from relapsing infections in mice treated with high doses, but were less common (Supplementary Fig. S12a). Furthermore, it is possible that the high-level resistance in some infections was conferred by sub-populations with alternative or additional mutations each of which existed at frequencies insufficient to feature in our consensus sequence of the Qo2 domain generated by Sanger sequencing. Indeed, we frequently detected evidence of mixed infections when we examined sequencing electropherograms, particularly in infections where high-level resistance emerged (Supplementary Fig. S12b). It is also possible that some levels of resistance (Fig. 3B) were conferred by mutations outside of the Qo2 domain. Mutations elsewhere in *cytb* are known to be associated with atovaquone resistance, although these tend to arise following treatment with low doses of atovaquone (lower than those tested here) and exhibit considerably reduced degrees of resistance [57, 62, 70]. These, however, may have contributed to conferring some degree of phenotypic resistance in our Qo2 wildtype populations (Supplementary Figs S3 and S12b). Alternatively, some degree of resistance may have been conferred by minority Qo2 resistant genotypes. Full and deep genome sequencing of the parasites that emerged in our experiments could be revealing for changes in the frequency of resistant mutants and the contribution of any non-Qo2 mutations to resistance.

In experiments 1 and 2, dose had contrasting impacts on the probability that parasites mutant at the Qo2 domain would emerge (Figs 2 and 3A). Dose had no impact in our first experiment, but in our second, the likelihood of mutations arising in the Qo2 domain increased with dose. There are several possible explanations for the differences between experiments, and while we did not explicitly test for it, differences in mouse strain seem most likely. It is well known that antimicrobials are less efficacious and resistance evolution is more likely in immune-impaired individuals [71–73], and indeed, parasite populations were smaller at the time of treatment and drug-induced clearance rates were greater in the more resilient C57BL/6 mice (experiment 1) than in Swiss Websters (experiment 2). Differences in the relative contribution of genetic drift and selection following drug treatment or in the relationship between dose and resistance may account for this. Our data support further analysis of host heterogeneity, especially immune heterogeneity, as a determinant of optimal dosing strategies for antimicrobial therapies.

Our results differ from many *in vitro* studies and a few *in vivo* studies in which the evolution of resistance was reduced at higher doses [74–76]. The discordance between these studies and ours likely arises from the existence of the so-called inverted U, where resistance evolution is minimized at very low and very high doses and maximized somewhere in-between [3, 4]. Presumably, if we had increased concentrations still further, we would have reached a point where there were no mutants capable of surviving and so no resistance would have emerged despite the intense selection. However, with our formulation, we were up against the upper bound of the therapeutic window, the point at which the drug itself becomes harmful. Atovaquone has limited solubility (even in DMSO) and the solvent itself is toxic [77], particularly in mice already suffering from malaria infection (data not shown). It is possible that higher doses, perhaps delivered via oral administration, would reveal the full inverted U. Nonetheless, our data caution against the simple mantra that more is better. Even though the inverted U is well understood in theory and well verified *in vitro*, determining whether a low or high dose strategy is most appropriate will depend on the effects of each option on host health and on considerations of the drug, its formulation and its pharmacokinetics and dynamics [4]. The right dose need not be the highest dose [3, 4, 24]. In our case in particular, increasing dose did not result in faster parasite clearance (at least at levels detectable by qPCR) and provided no increasing benefit with respect to survival. But it did strongly promote resistance evolution.

### Timing and the emergence of resistance

When the timing of treatment was altered, infections treated closest to the time of peak parasite density were more likely to result in resistance (here treated only as binary genotypic resistance). As such, treating early or very late in infection resulted in decreased probabilities of resistance emergence (experiment 3 and 4, respectively; Fig. 4A and C). The simplest explanation for the effect of timing on resistance evolution is the size of the pathogen population at the time of treatment: population sizes are small early and late in infection (Fig. 4B and D), consistent with the general principle that larger population sizes are more likely to contain drug-resistant mutations on which selection can act [40–43]. However, for infections treated after peak densities (experiment 4), an alternative explanation is that developing immune responses decrease the opportunity for resistance emergence. Models of pathogen growth in rodent hosts have shown that pathogen dynamics are disproportionately regulated by resource limitation (e.g. red blood cells) early in infections, but after 10 days immune regulation dominates [78]. Relapses may thus be substantially restricted by active immunity when treatment is given later. Post-treatment relapses were indeed smaller when treatment began on day 12 post-infection than those following treatment on days 6 and 9 post-infection (Fig. 4C). Parasite clearance was not obviously predictive of resistance, however, as parasites were cleared with similar rapidity on days six and seven post-infection as during late in infection in experiment 4 (Supplementary Fig. S9a). Ideally, the effect of timing on both pre- and post-peak densities would be tested in a single mouse strain to confirm these findings.

Even if immunity restricts resistance emergence later in infections, our data suggests that when the parasite population is expanding, the earlier treatment occurs, the better (experiment 3, Fig. 4A). The most likely explanation for this is the smaller populations at the time of treatment. This seems even more likely given that parasite clearance earlier in infection in experiment 3 was less efficient than later on in infection (Supplementary Fig. S9a). We directly tested this by altering the initial inoculum size to generate different populations on a fixed day of treatment (experiment 5). We found that resistance evolution was maximized at intermediate inoculum sizes (10 000 parasites, Fig. 4E). While we expected that increasing inoculum sizes would result in concurrent increases in parasite population size at the time of drug treatment, they were similarly maximized at intermediate inoculum sizes, with the largest population sizes generated by an inoculum size of 10^6^ (Fig. 4F). The reason for this is unclear, and may reflect some biases in our experimental set-up. Indeed, only three of eight mice survived the acute-stage of infection in our highest inoculum size manipulation (10^7^). As such, the mice which we included in our analysis may reflect mice that received a lower inoculation by chance or instances in which mice were able to better control their parasite populations. Despite this, our data suggest that the risk of resistance was more related to population size than inoculum size. However, resistance risk did not differ between mice given an inoculum size of 10 000 or 10^6^ parasites, irrespective of significant population size differences (log10 population size–experimental group, *F*_1,13_= 169.6, *P* < 0.001, Fig. 4E; resistance–experimental group, *F*_1,13_= 2.4, *P* = 0.15, Fig. 4E).

Taken together, this suggests that the relationship between population size and resistance is not altogether straightforward, or that its effect may be overwhelmed by other factors when differences in population sizes are small or moderate. We found a correlation between resistance emergence and population size in each of our three experiments (Supplementary Fig. S8), but we cannot statistically disentangle this from the effects of our experimental treatments *per se* (Supplementary Table S1). This might be a detection issue. Manipulating inocula sizes did not generate populations as small as those in the other experiments (Fig. 4B, D, F and **Supplementary Fig. S7**), and that more limited between-group variation may have been insufficient to reveal a clear relationship between population size and resistance risk. Likewise, in all three experiments, the variation in population size within experimental manipulations was small relative to the between group variation (Fig. 4B, D, F), again perhaps limiting our ability to detect an effect.

Further experiments are required to resolve this. In the meantime, all we can say is we do not have direct experimental evidence that treating early prevents resistance emergence because populations are small, even though that seems the most obvious explanation.

### ‘Right’ dose, ‘right’ timing?

Our experiments are some of the very few that directly measure *de novo* resistance emergence *in vivo* as a function of treatment regimen. What do they say about the ‘right’ dose and ‘right’ timing of patient treatment? Our aim was to advance the science of regimen choice, and we cannot expect to identify particular regimens that will be optimal in real-world settings: details matter [4]. Nevertheless, we can ask for our animal model, what dose and timing best manage resistance emergence while simultaneously maximizing host health, and we can consider the implications were those findings to generalize.

We found unequivocal evidence that the timing of treatment matters (Fig. 4A and C), so that from the perspective of resistance management within a host it is better to treat as early as possible or to withhold treatment until immunity has built up. This accords with theory [34, 36, 79] and the long standing ‘hit-early’ orthodoxy [80]. The practicalities of hitting early (or late) will depend on the circumstances. It may be that once symptoms are detected, infections are already advanced to the point where resistance emergence is a real risk. The feasibility of withholding treatment until immunity is building will be very situation specific. In our model, withholding treatment is only an option in resilient C57BL/6 mice because acute infection is often lethal for Swiss Webster mice if treatment is delayed. However, even in C57BL/6 mice, the choice of late treatment would need to be balanced against the potential benefits to host health of early treatment.

The ‘right’ dose differed between experiments. In our two experiments where we varied drug concentration, atovaquone dose affected resistance emergence (Figs 2 and 3) yet benefits to host health were minor, with no significant differences in anemia during acute or chronic stages of infection (Supplementary Fig. S4c and d). In our first experiment, however, increasing drug dose resulted in fewer treatment failures overall (Fig. 2A), and as relapses resulted in greater anemia later in infection, there is an argument that the highest doses were the ‘right’ dose, despite the consequences for resistance. In our second experiment where increasing dose did not decrease the probability of treatment failure, it would seem the best option is to use a low dose to allow the possibility of further treatment during relapse. These contrasting conclusions arising in even our highly simplified setting demonstrate that identifying the ‘right’ dose is a non-trivial problem.

Minimally, then, our data demonstrate the need for continued evaluation of what constitutes ‘appropriate’ antimicrobial treatment [3, 4]. Many questions remain. For example, the impact of host immunity on pathogen dynamics during drug treatment is a big knowledge gap [33, 81]. This is especially important as current searches for new antimicrobials continue to focus on fast killing compounds [82] and drug development pipelines make little room for testing evolution of resistance in *in vivo* scenarios.

## SUPPLEMENTARY DATA

Supplementary data is available at *EMPH* online.

## Supplementary Material

eoaa016_Supplementary_Data
