# Supplementary material for: Effect of drug dose and timing of treatment on the emergence of drug resistance in vivo in a malaria model
Source: Evol Med Public Health. 2020 Jun 1;2020(1):196–210. doi: 10.1093/emph/eoaa016 (PMC7652304; doi:10.1093/emph/eoaa016)
Supplement: eoaa016_Supplementary_Data [file eoaa016_supplementary_data.pdf]

## SUPPLEMENTARY TEXT

### *Sequencing relapsed infections and quantifying mixed infections (experiment 1 and 2)*

Parasite DNA from samples at peak relapse were amplified using forward: 5' TCC TTT AGG GTA TGA TAC AGC 3' and reverse primers: 5' CTT GAG GTA ATT GAC ATC CTA TC 3', at a final concentration of 0.3 μM using the Qiagen *Taq* PCR Core Kit and purified via the Qiagen QIAquick PCR Purification Kit prior to sequencing. To confirm that the initial parasite populations from which we selected resistance were indeed wildtype at the Qo2 region, we amplified and sequenced parasite DNA from twenty infections in experiments 1 and 2 on the first day of drug treatment. Only the wildtype genotype was detected. We also sequenced parasites from untreated infections later in infection (ten mice in experiment 1 on day 13 post-infection and a single mouse in experiment 2 on day 20 post-infection) and detected only the wildtype Qo2 genotype. Thus, in the absence of drug treatment, any mutations in that region were at densities below our detection threshold.

In almost all cases sequencing of the amplicon in both the forward and reverse direction resulted in identical sequences, however, a minority of samples differed with respect to either the majority genotype represented or in the presence of mixed infections detected. In all main text analyses, we refer to the majority genotype, obtained from Sanger sequencing in the forward direction. In our analyses here, we consider the presence of mixed infections, defined as: infections in which forward and reverse sequences differed and/or and minority peaks were detected in sequencing (at least 25% peak similarity, as detected by Geneious® version 9.1.8 and confirmed via manual inspection).

Mixed infections were common within our relapsing populations. We re-classified sequences from infections into three categories: wildtype (no detection of minority peak alleles), mutant (no detection of minority peak alleles) and mixed (evidence of minority peak alleles); based on both the forward and reverse sequences. Data from our phenotypic measures of resistance suggested that resistance phenotype varied depending on genotype in experiments 1 and 2 (genotype: $F_{9,55} = 6.6$, $p < 0.001$, **Supplementary Fig. S12b**). We found that resistance phenotype was determined in part by the presence or absence of mixed infections. Wildtype only infections were found to have the lowest slopes of parasite growth in the presence of drug in naïve mice (0.04 +/- 0.19, 95% confidence interval), followed by mixed infections (0.58 +/- 0.09, 95% confidence interval) and finally mutant only infections (0.62 +/- 0.10, 95% confidence interval).

The large spread in phenotypic variance for wildtype genotypes is likely because some of these relapsing populations contained sub-dominant clones. Given the low ability of Sanger sequencing to fully resolve mixed infections, it is likely that our estimations of mixed infections are highly conservative and that drug treatment resulted in relapse with highly diverse pathogen populations that differed in genotype and relative frequency in most cases. Selection here thus occurs as a soft selection sweep involving multiple and independent origins of the same or related alleles that confer drug resistance. Soft sweeps have been theorized to be common under scenarios of high mutation rates and/or high selection coefficients[1,2] and have even been predicted to occur given the complex biology of mitochondrially encoded atovaquone resistance[3].

**SUPPLEMENTARY REFERENCES**


1. Hermisson, J. Soft Sweeps: Molecular population genetics of adaptation from standing genetic variation. *Genetics* **169**, 2335–2352 (2005).
2. Hermisson, J. & Pennings, P. S. Soft sweeps and beyond: Understanding the patterns and probabilities of selection footprints under rapid adaptation. *Methods Ecol. Evol.* **8**, 700–716 (2017).
3. Cottrell, G., Musset, L., Hubert, V., Le Bras, J. & Clain, J. Emergence of resistance to atovaquone-proguanil in malaria parasites: Insights from computational modeling and clinical case reports. *Antimicrob. Agents Chemother.* **58**, 4504–4514 (2014).

## SUPPLEMENTARY TABLES

**Supplementary Table S1. GLM models of relapse and resistance.** Effects of atovaquone dose on relapse and resistance for experiment 1 and 2. Relapse is defined as sustained parasite growth following drug treatment. Resistance is defined as a relapse dominated by mutations in the Qo2 region of the *cytb* gene (see main text for further details). For experiments 3-5, we tested significance of experimental manipulation on relapse and resistance and then determined whether adding population size accounted for any additional explanatory power via likelihood testing as indicated.

**Relapse (treatment failure):**
Full model (relapse for experiments 1 and 2): relapse ~ log10(dose)
Full model (relapse for experiments 3 to 5): relapse ~ experimental group (5 or 6 levels depending on experiment) + log10(population size at time of treatment)

| Experiment | Model term | d.f. for term | Relationship | $\chi^2$ | P value |
|---|---|---|---|---|---|
| Exp. 1 | dose | 1 | Negative | 7.11 | **0.01** |
| Exp. 2 | dose | 1 | - | 1.20 | 0.27 |
| Exp. 3 | experimental group | 4 | - | 27.61 | **<0.001** |
| | + log10(population size) | 1 | - | 0.52 | 0.47 |
| Exp. 4 | experimental group | 5 | - | 29.62 | **<0.001** |
| | + log10(population size) | 1 | - | 1.27 | 0.26 |
| Exp. 5 | experimental group | 5 | - | 18.32 | **0.003** |
| | + log10(population size) | 1 | - | 1.96 | 0.16 |

**Resistance:**
Full model (experiments 1 and 2): resistance ~ log10(dose)
Full model (experiments 3 to 5): resistance ~ experimental group (5 or 6 levels depending on experiment) + log10(population size at time of treatment)

| Experiment | Model term | d.f. for term | Relationship | $\chi^2$ | P value |
|---|---|---|---|---|---|
| Exp. 1 | dose | 1 | - | 0.00 | 0.97 |
| Exp. 2 | dose | 1 | Positive | 19.71 | **<0.001** |
| Exp. 3 | experimental group | 4 | - | 27.61 | **<0.001** |
| | + log10(population size) | 1 | - | 0.52 | 0.47 |
| Exp. 4 | experimental group | 5 | - | 24.33 | **<0.001** |
| | + log10(population size) | 1 | - | 0.03 | 0.87 |
| Exp. 5 | experimental group | 5 | - | 15.23 | **0.01** |
| | + log10(population size) | 1 | - | 0.01 | 0.93 |

**Supplementary Table S2. Consensus haplotypes at the Qo2 domain of the *cytb* gene during relapses**. Tabulated values are the number of infections. Underlined amino acids in the first column denote mutated amino acids as compared to the wildtype sequence.

| Haplotype | Mutations | Previously reported* | Total Numbers | Total Numbers |
|---|---|---|---|---|
| | | | ***Experiment 1 & 2*** | ***Experiment 3-5*** |
| FYAMLK (wildtype) | - | - | 29 | 12 |
| FCAMLK | Y268C | yes[a,c,d,e,f] | 29 | 5 |
| FYAMVR | L271V + K272R | yes[a,c,e,f] | 18 | 12 |
| FNAMLK | Y268N | yes[b,c,e,f] | 7 | 10 |
| FSAMLK | Y268S | yes[d,f,g] | 5 | - |
| FYAMVK | L271V | yes[t] | 5 | 3 |
| IYAMLK | F267I | yes[a] | 4 | - |
| FYAMLR | K272R | yes[t] | 3 | 3 |
| FCAMVK | Y268C + L271V | yes[t] | 1 | - |
| VYAMLK | F267V | no | 1 | 2 |

[*] column indicates study in which mutations were reported: [a] Srivastava *et al* 1999 (*P. yoelii)* , [b] Afonso *et al* 2010 (*P. chabaudi*), [c] Siregar *et al* 2008 (*P. bergheii*), [d] Musset *et al* 2006, [e] Nuralitha *et al* 2017, [f] Nuralitha *et al* 2016, [g] Korsinczky *et al* 2000. [t] denotes mutations where mutation has been previously reported, but not in the specific haplotype indicated here.

## SUPPLEMENTARY FIGURE CAPTIONS

**Supplementary Fig S1. Five species alignment of the *cytochrome b* gene from malaria parasites.** Sequences of the full-length *cytochrome b* gene are shown for a representative atovaquone sensitive (wildtype) strain of *P. falciparum* and *P. vivax* (human infecting species; PlasmoDB reference IDs: Pf_M7661101900.1, PVAD80_MIT0003.1, respectively) and *P. yoelli, P. chabaudi* and *P. berghei* (rodent infecting species; PlasmoDB reference IDs: PYYM_MIT00900.1, PCHAS_MIT01800.1, PBANKA_MIT01900.1, respectively). Sequences were aligned via Geneious version 9.1.8. Sequence homology is represented above the alignment with green bars, indicating large levels of conservation even among these distantly related species. Blue arrows indicate positions of our forward and reverse primers used in sequence amplification and sequencing. Red bars indicate known and reported mutations associated with resistance to atovaquone in previously reported *in vitro* and *in vivo* studies. Black arrows indicate the quinone binding 2 (Qo2) region of the gene in which high level resistant mutations have been reported. The red box indicates where all the presently reported mutations were located.

**Supplementary Fig S2. Estimating population sizes at time of treatment for early treatment groups in experiment 3**. We fit a 3-parameter logistic model to data from untreated mice during parasite growth following inoculation of an estimated 100 parasites during days five to 12 post infection. In blue is data from our untreated mice, while red points are estimates for parasite numbers on days when parasites were below the level of detection in our qPCR assay (dotted line), as predicted by model fit.

**Supplementary Fig S3. Growth rates of Qo2 mutant** (red) **and wildtype** (blue) **parasites in naïve drug-treated mice**.

**Supplementary Fig S4. Effects of atovaquone dose on parasite dynamics and host health**. Effects of dose or experiment are shown for experiments 1 (black) and 2 (purple) in each panel. **(a)** Rate of parasite clearance during and immediately after drug treatment (days 6-10 post-infection). **(b)** Average population sizes at time of treatment**.** (**c,d**) Minimum red blood cell densities for individual mice during acute (**c**) and chronic (**d**) stages of infection. Only data from relapsed mice are shown for the chronic stage of infection. Dotted black lines separate drug treated and control untreated mice. Error bars denote 95% confidence intervals.

**Supplementary Fig S5. Kaplan-Meier survival curves for experiments 1 and 2**. Survival curves are shown for mice in experiment 1 (**a**) and 2 (**b**). Unbolded p-values represent the effect of all experimental groups on survival probability; *Bold p-values when omitting no-drug controls.

**Supplementary Fig S6. Average red blood cell dynamics for experiments 1 and 2.** Average red blood cell dynamics are shown through time for experiment 1 (**a**) and experiment 2 (**b**) for untreated (black), relapsed (blue) and non-relapsed infections (yellow).

**Supplementary Fig S7. Parasites per mouse at start of atovaquone treatment for experiments 3-5**. Horizontal bold lines show the mean, and boxplots show variance of population sizes generated by each experimental manipulation. $\sigma^2 = 5.81$ for experiment 3, $\sigma^2 = 1.17$ for experiment

4 and $\sigma^2 = 0.92$ for experiment 5 via generalized least squares model allowing for variance structure.

**Supplementary Fig S8. Population size at time of treatment and resistance emergence.** Parasite population size at the time of treatment plotted against the probability of resistance emergence across all experimental groups in experiments 3 (red), 4 (blue) and 5 (yellow). Fitted curves reflect logistic model predictions fitted for each experiment.

**Supplementary Fig S9. Effects of treatment timing or inoculum size on parasite clearance and host health.** Effects of experimental manipulation are shown for experiments 3 (red), 4 (blue) and 5 (yellow) in each panel. **(a)** Rate of parasite clearance calculated by fitting a linear slope to data from individual mice on day of, and three days post treatment. **(b,c)** Minimum red blood cell densities for individual mice during acute (**b**) and chronic (**c**) stages of infection. Data from relapsed and non-relapsed are shown separately in **c**, but statistics represent differences across experimental manipulations, irrespective of relapse. Error bars denote 95% confidence intervals.

**Supplementary Fig S10. Kaplan-Meier survival curves for experiments 3-5**. Survival curves are shown for mice in experiment 3 (**a**), 4 (**b**) and 5 (**c**). P-values represent the effect of experimental groups on survival probability, fit as discrete groups.

**Supplementary Fig S11. Average red blood cell dynamics for experiments 3, 4 and 5.** Average red blood cell dynamics are shown through time for experiment 3 (**a**), experiment 4 (**b**) and experiment 5 (**c**) for mice which died during the acute stage of infection (red), relapsed (blue) and non-relapsed infections (yellow). Astericks denotes a day on which data from red blood cell counts were lost from experiment 5 and thus not included.

**Supplementary Fig S12. High-level resistance is associated with a diversity of mutations.** (**a**) The proportion of relapses due to a specific genotype are shown across dose for data combined between experiments 1 and 2. (**b**) Parasite growth of relapsed infections in atovaquone treated tester mice is shown for experiments 1 and 2 as a function of their dominant genotype. Colors show whether or not evidence of mixed infections were found for each individual mouse relapse (black indicating mixed infection). Blue is wildtype drug-sensitive with no evidence of mixed infection, red is mutant drug-resistant with no evidence of mixed infection.

**SUPPLEMENTARY FIGURES**

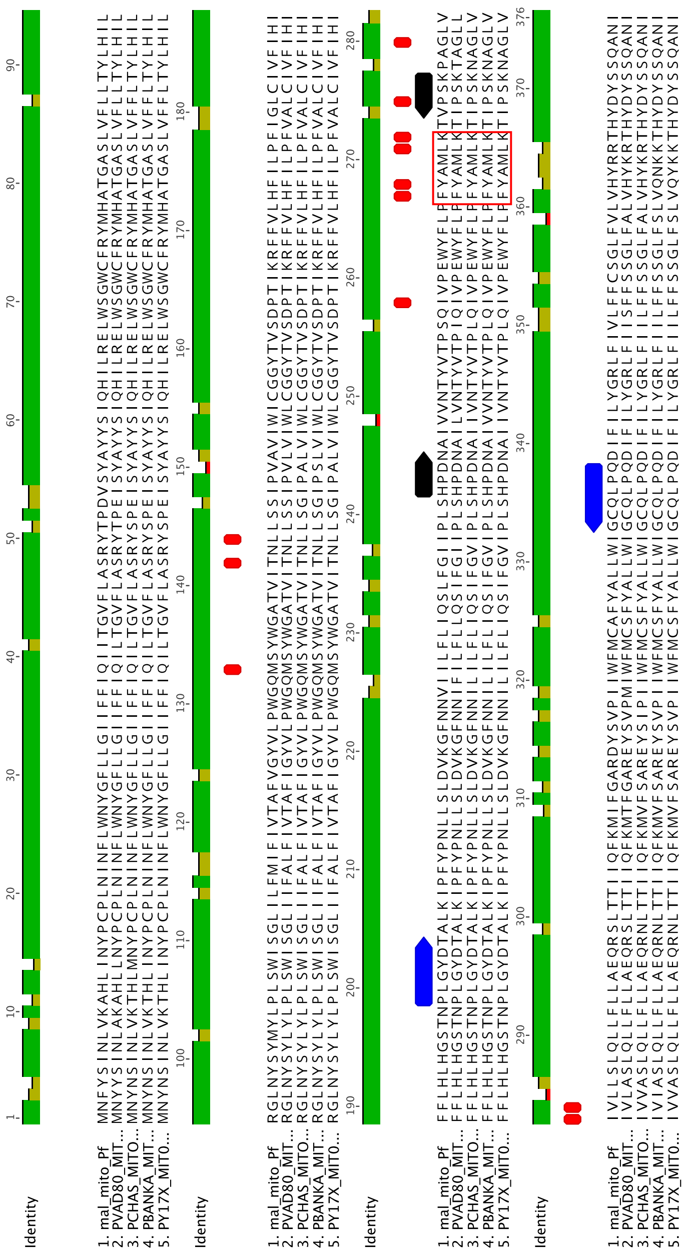


**Supplementary Fig S1.**

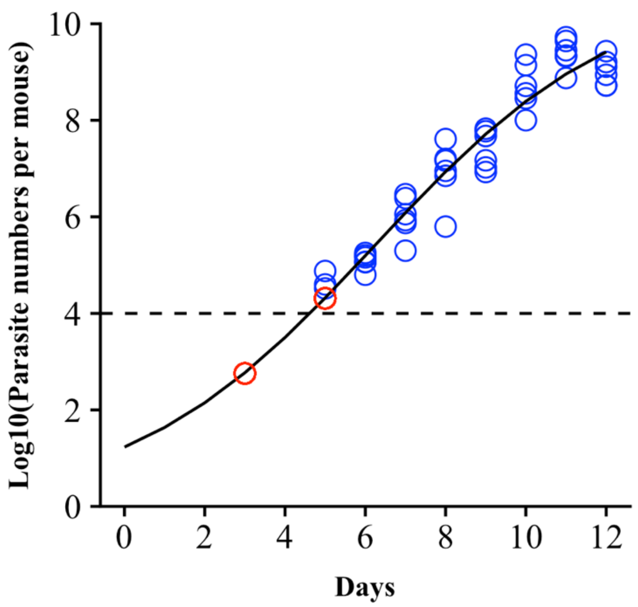


**Supplementary Fig S2.**

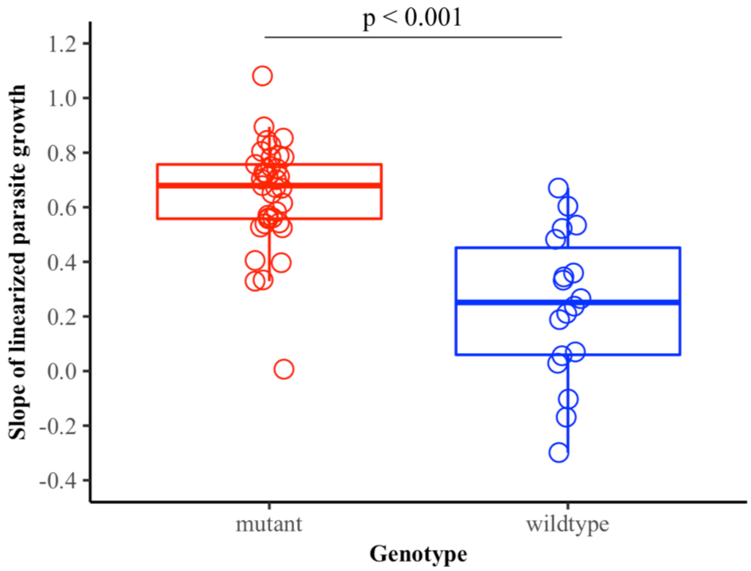


**Supplementary Fig S3.**

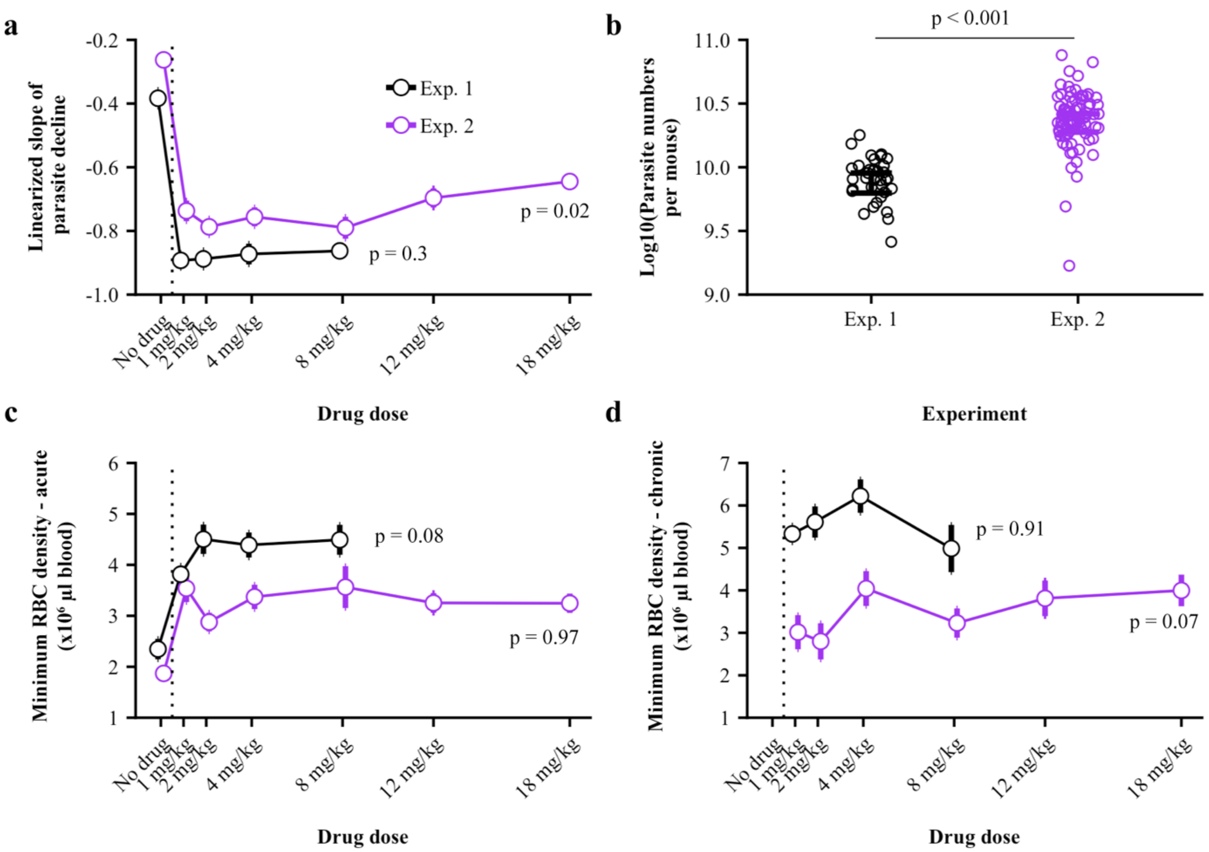


**Supplementary Fig S4.**

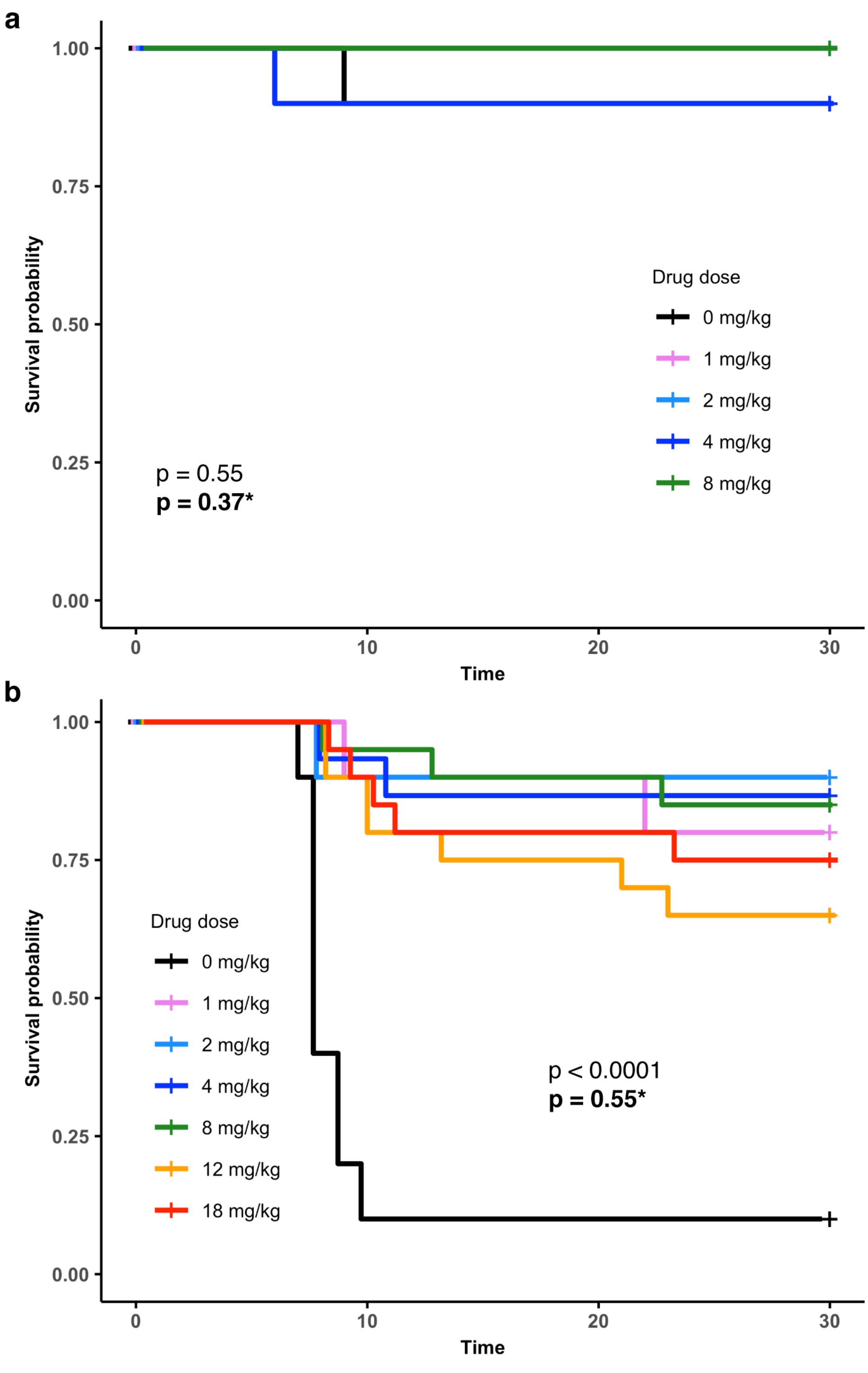


**Supplementary Fig S5.**

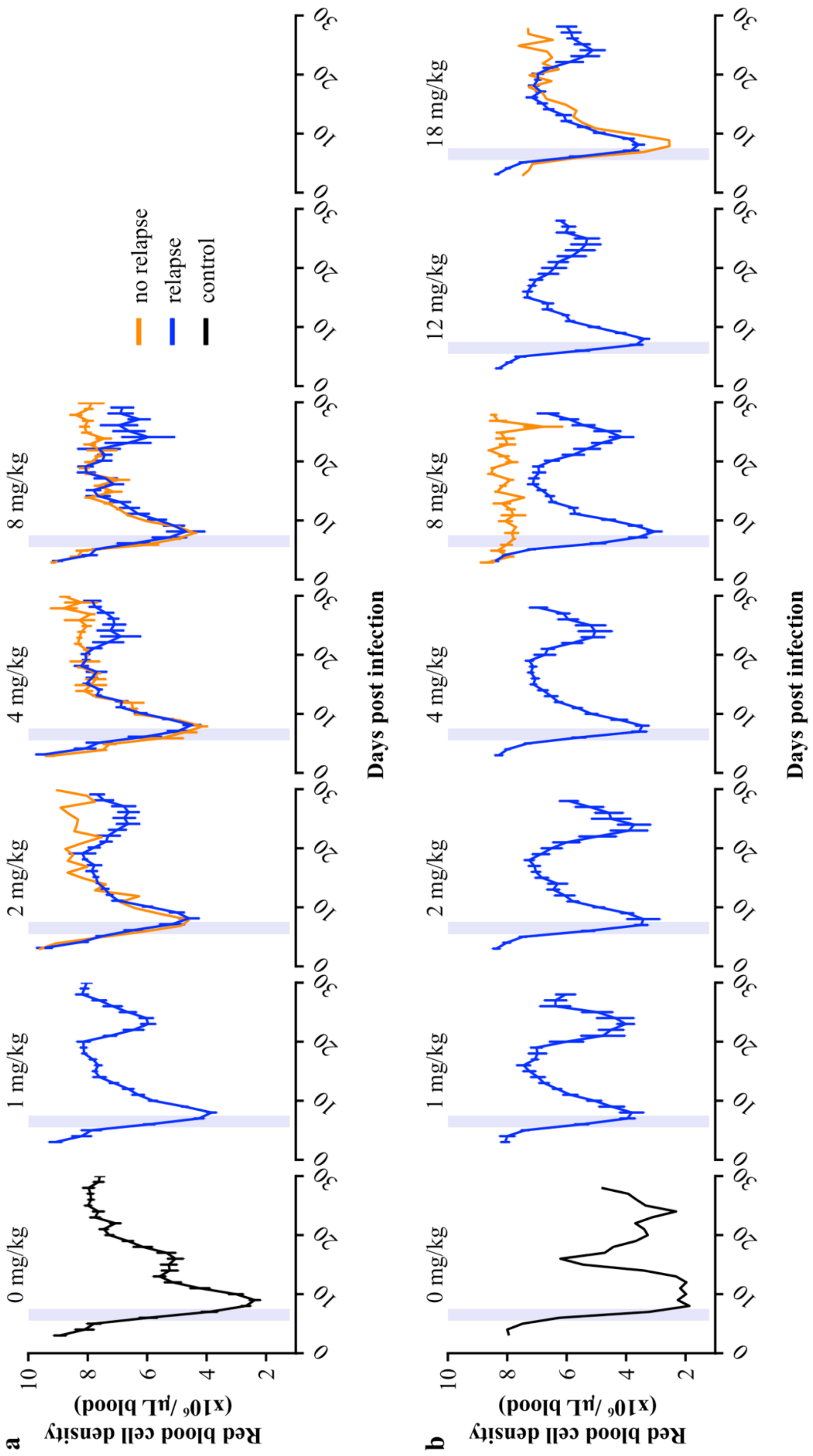


**Supplementary Fig S6.**

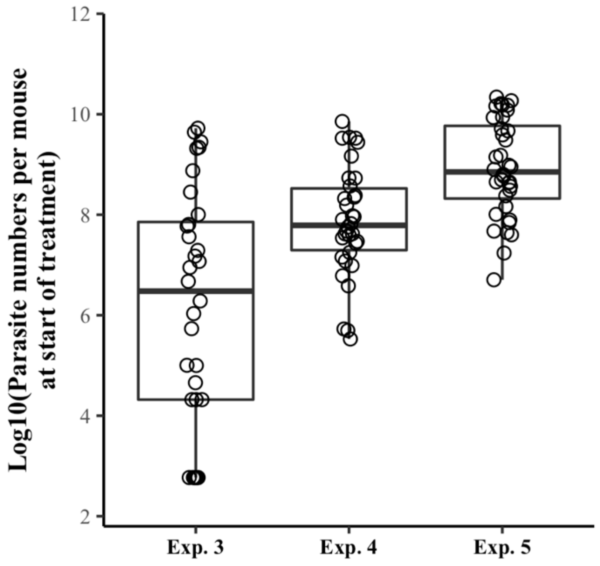


**Supplementary Fig S7.**

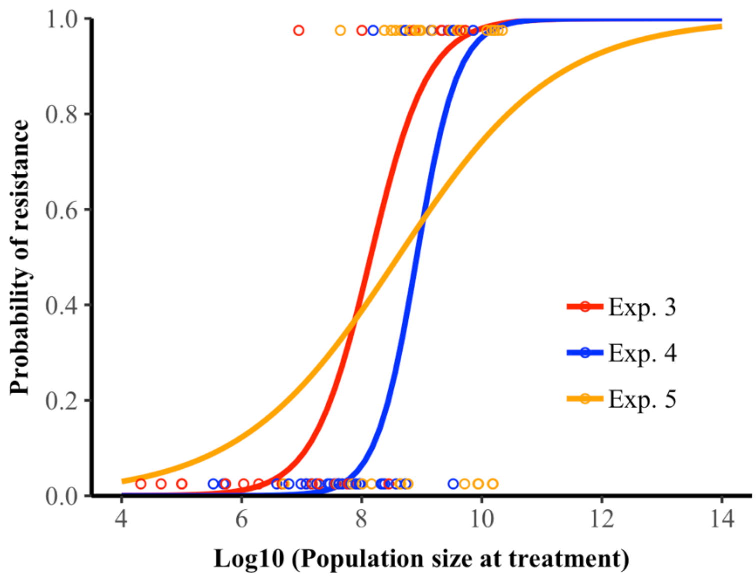


**Supplementary Fig S8.**

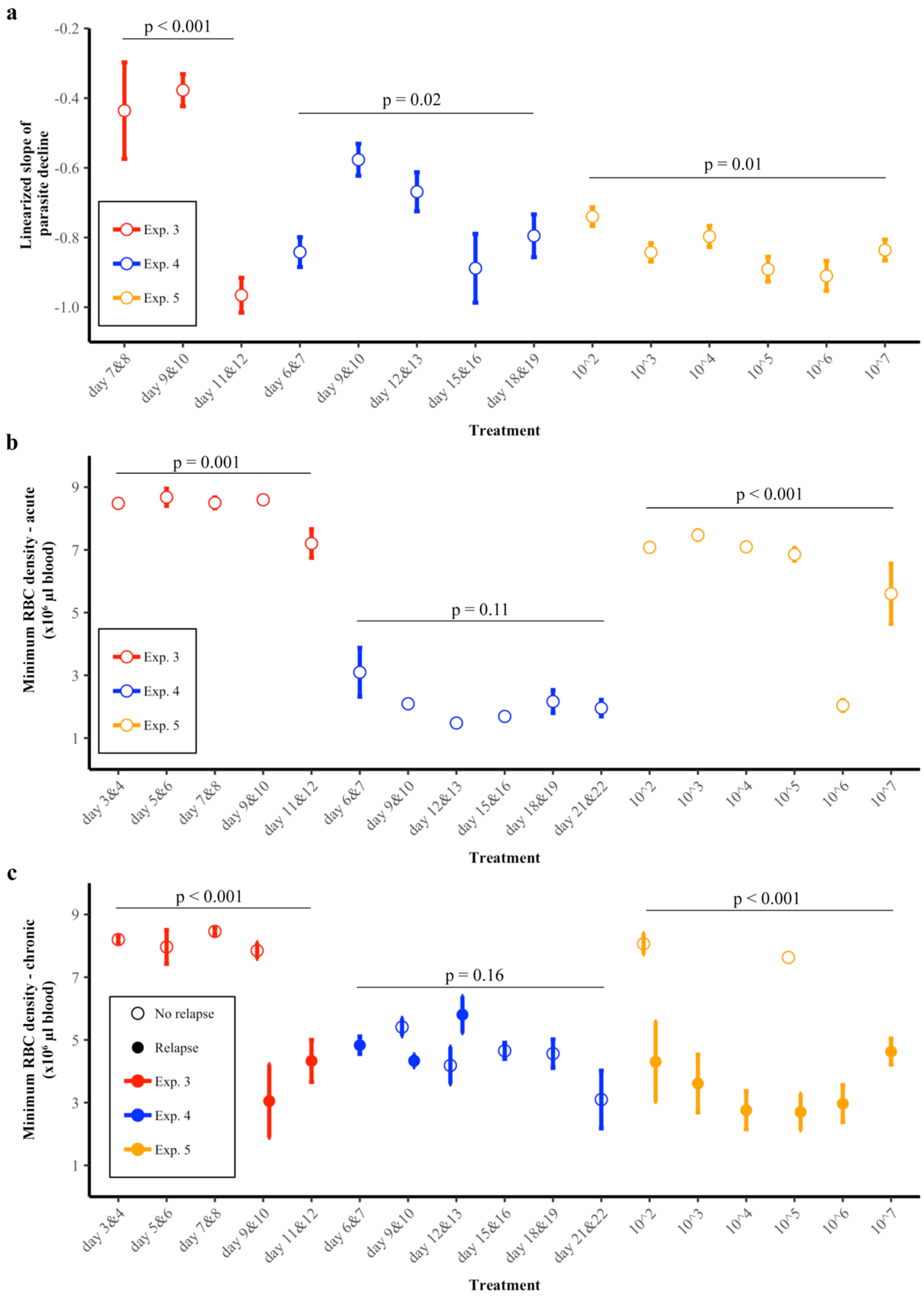


**Supplementary Fig S9.**

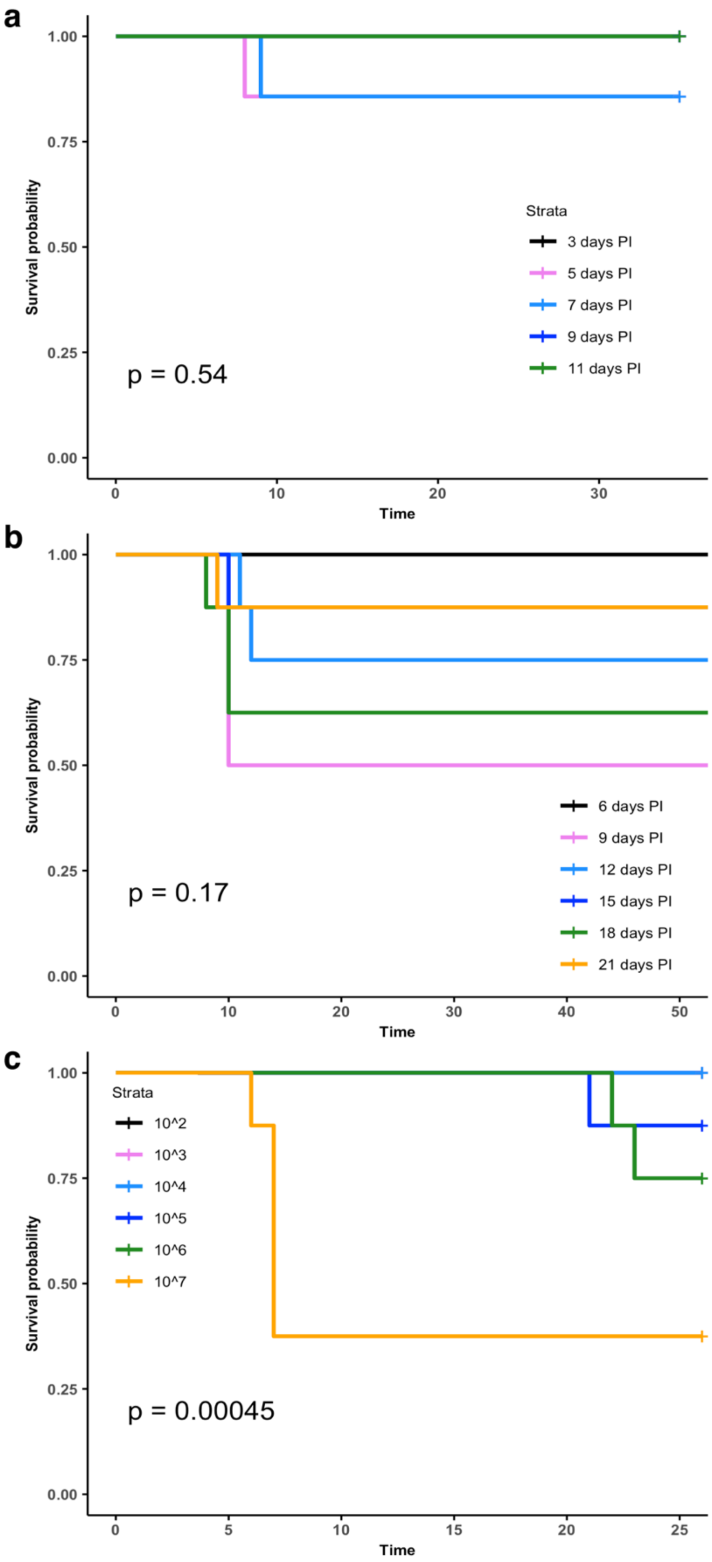


**Supplementary Fig S10.**

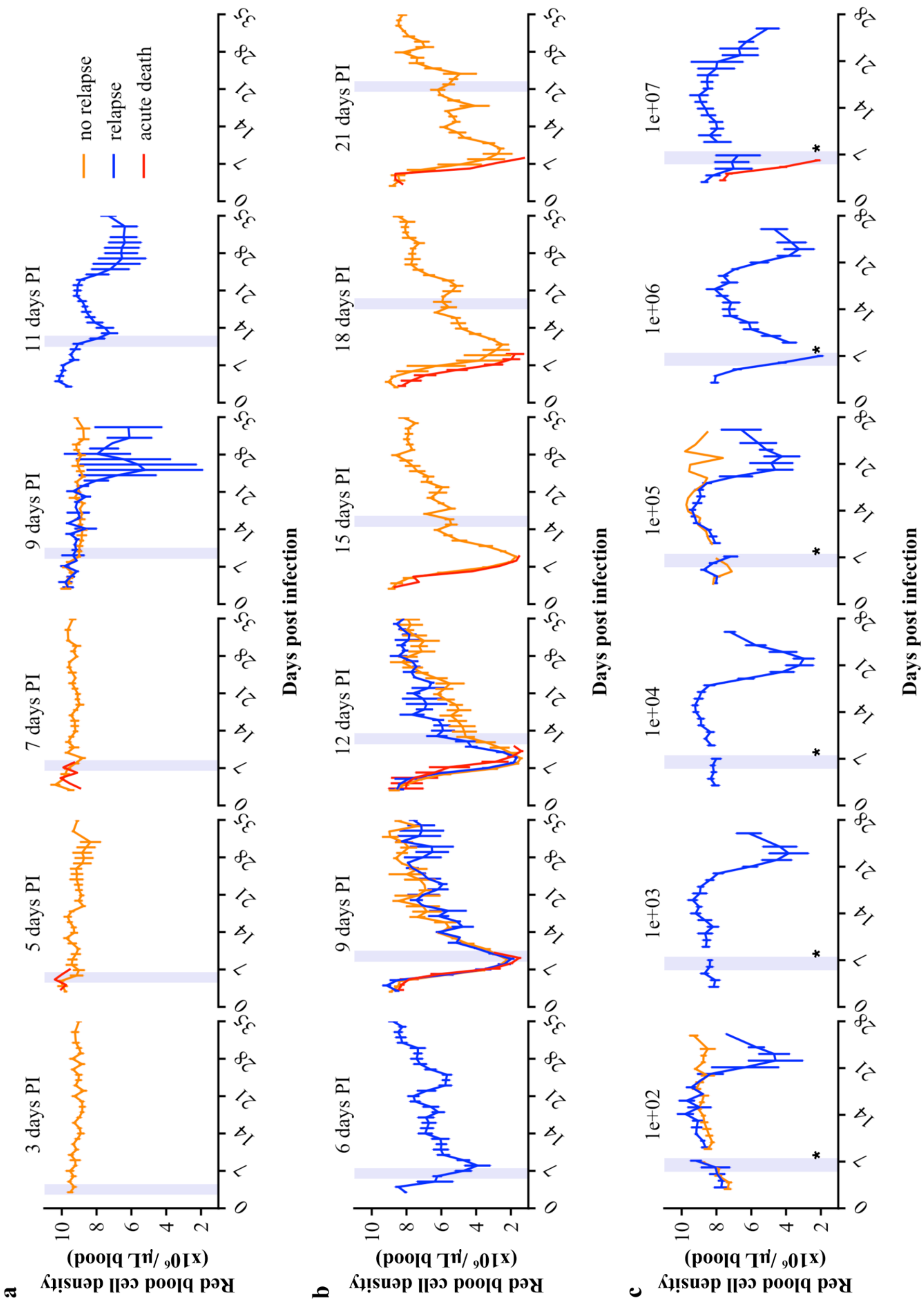


**Supplementary Fig S11.**

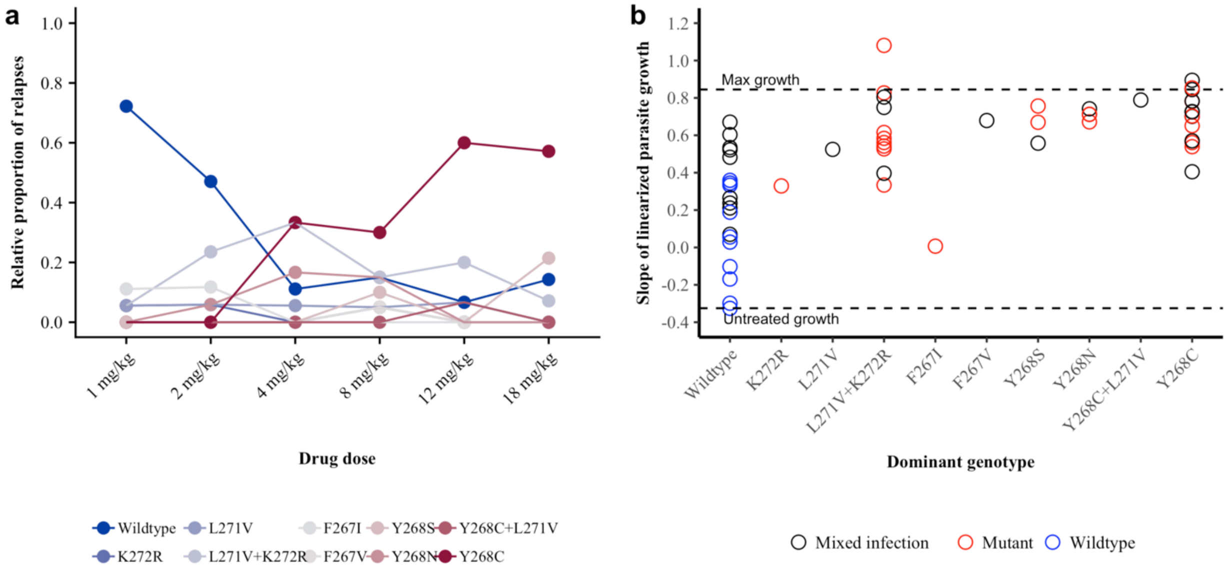


**Supplementary Fig S12.**
